# Transcriptional profile of a bioethanol production contaminant *Candida tropicalis*

**DOI:** 10.1186/s13568-018-0693-1

**Published:** 2018-10-11

**Authors:** Natália Manuela Strohmayer Lourencetti, Ivan Rodrigo Wolf, Maria Priscila Franco Lacerda, Guilherme Targino Valente, Cleslei Fernando Zanelli, Mariana Marchi Santoni, Maria José Soares Mendes-Giannini, Francisco Javier Enguita, Ana Marisa Fusco-Almeida

**Affiliations:** 1 São Paulo State University (UNESP), School of Pharmaceutical Sciences, Araraquara, São Paulo Brazil; 2 School of Agromonic Sciences, São Paulo State University (UNESP), Botucatu, São Paulo Brazil; 3 School of Medicine Science, Lisbon University, Lisbon, Portugal

**Keywords:** Alcoholic fermentation, Bioethanol, *Candida tropicalis*, Contaminant, RNA-seq

## Abstract

The fermentation process is widely used in the industry for bioethanol production. Even though it is widely used, microbial contamination is unpredictable and difficult to control. The problem of reduced productivity is directly linked to competition for nutrients during contamination. Yeasts representing the *Candida* species are frequently isolated contaminants. Elucidating the behavior of a contaminant during the fermentation cycle is essential for combatting the contamination. Consequently, the aim of the current study was to better understand the functional and transcriptional behavior of a contaminating yeast *Candida tropicalis*. We used a global RNA sequencing approach (RNA-seq/MiSeq) to analyze gene expression. Genes with significantly repressed or induced expression, and related to the fermentations process, such as sugar transport, pyruvate decarboxylase, amino acid metabolism, membrane, tolerance to high concentrations of ethanol and temperatures, nutrient suppression), and transcription-linked processes, were identified. The expression pattern suggested that the functional and transcriptional behavior of the contaminating yeast during fermentation for bioethanol production is similar to that of the standard yeast *Saccharomyces cerevisiae*. In addition, the analysis confirmed that *C. tropicalis* is an important contaminant of the alcoholic fermentation process, generating bioethanol and viability through its tolerance to all the adversities of a fermentation process essential for the production of bioethanol. According on the gene expression profile, many of these mechanisms are similar to those of *S. cerevisiae* strains currently used for bioethanol production. These mechanisms can inform studies on antimicrobials, to combat yeast contamination during industrial bioethanol production.

## Introduction

Sugarcane is widely distributed in the tropical and subtropical regions, and is the main source of sugar and bioethanol (Amorim et al. 2011). For example, an estimated production of 691 billion tons of sugarcane from the 2017/18 crop and 27 billion liters of ethanol has been reported for Brazil, according to the Campanha Nacional de Abastecimento (National Supply Campaign) (CONAB 2018). The sugarcane used for bioethanol production is derived from *Saccharum* ssp., with hybrids from crosses between *Saccharum officinarum* and *Saccharum spontaneum* used in the industry (Dillon et al. 2007; Canilha et al. 2012). The most commonly used yeast in the sugarcane industry is *S. cerevisiae*, because of its ability to adapt to the various growth conditions during bioethanol production (Liti et al. 2009). The first-generation process for bioethanol production is widespread in Brazil, but it involves non-sterile conditions, resulting in major microbial contamination (Basso et al. 2008).

Contaminations of the fermentation process may be of soil or plant origin, with bacteria, such as *Lactobacillus* ssp., and yeasts, such as *Candida* ssp., *Pichia* ssp., and *Schizosaccharomyces* ssp. (Cabrini and Gallo 1999). These contaminants can negatively impact the productivity of *S. cerevisiae* by competing for nutrients, and inhibiting its cellular metabolism by flocculation, toxin synthesis, and the production organic acids (Nobre et al. 2007; Skinner and Leathers 2004). Controlling contaminant growth and verifying their behavior in the presence of a variety of fermentative microorganisms is essential (David et al. 2014). We therefore aimed to elucidate the transcriptional behavior of a relevant yeast contaminant, *C. tropicalis*, during a fermentation cycle using high-throughput sequencing.

## Materials and methods

### Yeast strain

The yeast strain analyzed in the current study was *C. tropicalis* ATCC MYA3404 (Broad Institute, USA). It is one of the main soil contaminants in sugarcane plantations, and had been isolated from a fermenter tank in Brazil. Yeast cultures were maintained in a yeast extract-peptone-dextrose medium [YEPD; 2% dextrose w/v (Synth—Diadema, Brazil), 1% yeast extract w/v (Kasvi—Curitiba, Brazil), 2% peptone w/v (Acumedia—Indaiatuba, Brazil), and 2% bacteriological agar w/v (Himedia—West Chester, Pennsylvania, USA)] at 30 °C.

### Cyclic alcoholic fermentation

The cells were first cultured in the YEPD liquid medium to obtain an appropriate biomass (10%, w/v) for scaling up. For this, they were first grown in an Erlenmeyer flask with total volume of 50 mL, initially inoculated with 5 × 10^6^ cells/mL and then grown with orbital shaking at 150 rpm at 30 °C for 24 h. Then, the cultures were centrifuged at 3 g, the supernatant was discarded, the cell mass suspended in fresh YEPD liquid medium, and transferred to a new Erlenmeyer flask (250 mL), up to 20% of the total volume of the flask. The cultures were grown with orbital shaking (150 rpm) at 30 °C for 24 h. The cultures were similarly scaled up to Erlenmeyer flask volumes of 500 mL, 1 L, and 2 L, to finally obtain the desired amount of biomass for the fermentation test.

For the fermentation assay, 100 mL of sugarcane juice was pasteurized by boiling and sterilized in an autoclave at 1 atm 121 °C. With the sterilize sugarcane broth, 10 g of the *C. tropicalis* biomass was added and the fermentation proceeded with orbital shaking (50 rpm), at 30 °C for 8 h. Samples (30 mL) were collected at the begging and the end of fermentation, immediately after the inoculation (0 h), and after 8 h fermentation. These times were selected primarily because it is known that a fermentation peak of yeast occurs 6–8 h after the start of fermentation. They were also chosen to reveal, by monitoring the changes in expression of the functional and transcriptional genes, the strategy used by the contaminant yeast to adapt to, grow, ferment, and tolerate the changing culture conditions at the beginning and end of the fermentation, to compare with those of the standard yeast *S. cerevisiae*.

### RNA isolation and sequencing

The samples collected in replicate, yeast cells C0 and P0 (0 h), and C8 and P8 (8 h) were centrifuged at 3*g* at 4 °C for 10 min, and washed three times with PBS buffer. Total RNA from these two samples was isolated using RNeasy mini kit (QIAGEN—Hilden, Germany), according to the manufacturer’s protocol. The amount of RNA in each sample was determined using a Quibit fluorometer (Thermo Fisher Scientific—Waltham, Massachusetts, EUA), with the quality determined based on the RIN value and by visualization using a BioAnalyzer (Agilent Genomic—Santa Clara, CA, EUA). Purified total RNA was fragmented (fragments of approximately 76–200 bp) in a fragmentation buffer Illumina (San Diego, California, EUA). First-strand cDNA was synthesized using random hexamer primers. Double-stranded cDNA was purified using QuiaQuick PCR extraction kit (QIAGEN), which was followed by end-polishing. Sequencing adapters Illumina were added to the ends of RNA fragments, and the fragments were enriched by PCR amplification. Finally, the DNA library products were sequenced using an Illumina MiSeq platform.

### Differential expression and representative ontology terms

FastQC (http://www.bioinformatics.babharam.ac.uk/projects/fastqc/) was used for library analysis. The reads were filtered using Trimmomatic v 0.36 (Bolger et al. 2014), with the parameters “ILLUMINACLIP: adapters.fa:2:30:10, HEADCROP: 13, LEADING: 30, TRAILING: 26, SLIDINGWINDOWN: 4:22, MINLEN: 18”.

The reads were mapped to the *C. tropicalis* genome (accession number GCA_000006335.3, 2017/10/29) using HISAT2 (Kim et al. 2015) with default parameters. Read counts per million were generated using BEDTools Intersect v 2.25.0 (Quilan and Nall 2010) with default parameters, based on genome annotations. The differential gene expression analysis (C8/P8 vs. C0/P0) was performed using the DeSeq 2 package (Love et al. 2014). Genes with a false discovery rate (FDR) < 0.01 and log2 fold-change of 1 were considered to be differentially expressed. Gene ontology (GO) terms corresponding to *C. tropicalis* genes were obtained from the UniProt database, and an in-house Python v 3.5 script was used to determine the frequency of each term for the differentially expressed genes (DEGs) and were analyzed using REViGO (Supek et al. 2011). The Illumina sequencing data were deposited in the NCBI Sequence Read Archive database (SRA) under the accession number SRP150532 (ID: SUB4151841).

## Results

### Sequence data and mapping to the *C. tropicalis* genome

Sequence mapping resulted in an approximately 96% overall alignment to the *C. tropicalis* reference genome for the two samples, which represented two pairs of biological replicates (C0/P0 and C8/P8). Overall, 22 genes were excluded from further analysis because no reads have been mapped to them. Considering the number of clean reads in each sample, more reads were detected in the 0 h sample than the 8 h sample (Table 1).

**Table 1:** Quantification of the reads of the RNA-Seq (Illumina) sequencing and mapping of time 0 h and 8 h of samples of contaminant yeast, *C. tropicalis*

Sample name	Raw reads	Number reads filtering	Genome map (%)
C0 + P0	7,385,154	7,141,555	96.1
C8 + P8	6,486,114	6,261,749	96.2

Sequencing and mapping parameters of the C0 plus P0 and C8 plus P8 genome samples

### Differential expression analysis

Overall, 949 DEGs were identified (506 were up-regulated and 443 were down-regulated). As relevant to understanding, the pathways and pathways via used by *C. tropicalis* during the fermentation process, many of the top 100 genes (both up- and down-regulated) were essential for fermentation. These included alcohol dehydrogenase, pyruvate carboxylase, glycolysis/gluconeogenesis, sugar transport, meiosis, metabolism, stress tolerance (resistance), and cell structure. Interestingly, a large number of genes essential for fermentation were down-regulated, while genes involved in nuclear function, transcription, and tolerance were up-regulated (Table 2).

**Table 2:** Functional classification and quantification of differentially expressed gene at time 0 h and 8 h of contaminant yeast, *C. tropicalis*

Genes functions	Genes down regulated	Genes up regulated
Alcohol dehydrogenase	7	–
Sugar transport	9	6
Pyruvate decarboxylase acyl-coenzyme	5	1
Histone	1	4
Amino acid metabolism	15	17
Division cellular (meiosis)	1	1
Membrane	10	10
Metals transporter	3	2
Stress oxidative (resistance)	6	7
Glycolysis/gluconeogenesis	8	5
Nuclear (transcription)	9	19
Structure (wall)	3	–
Nucleotides	–	5
Hypothetical functions	19	27

Functional classification and quantification of down and up regulated and repressed genes from the C0 and P0 and C8/P8 samples. Statistical significant for Fold Change > 2 or < − 2 and *p* value < 0.01

**Fig. 1:**
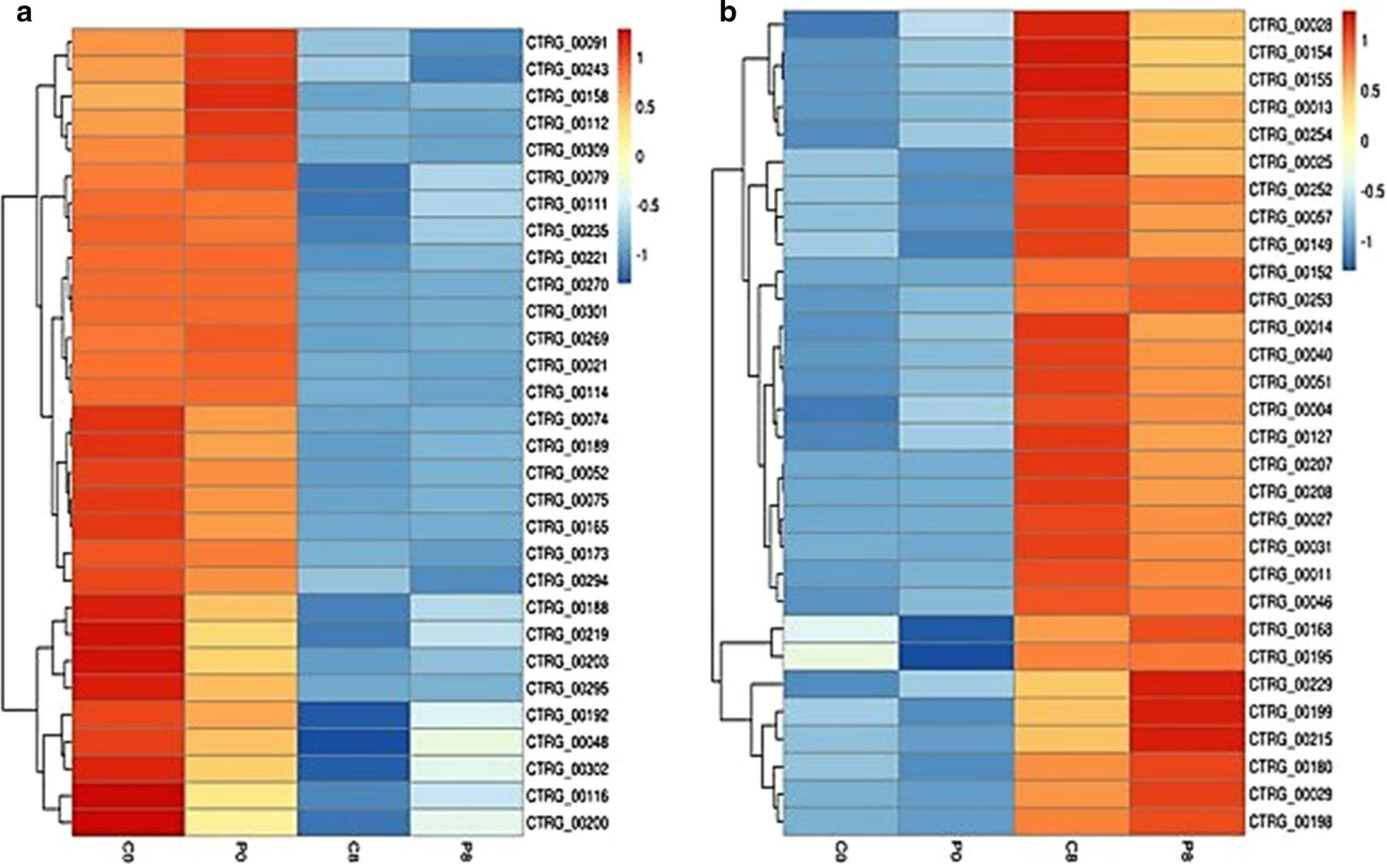
Heatmap of the top 30 differently expressed genes of contaminant yeast, *C. tropicalis* at 0 h and 8 h in the fermentation alcoholic cycle. **a** Down-regulated genes at 8 h (C8 and P8) in contrast to 0 h (C0 and P0); **b** up-regulated genes at 8 h (C8 and P8) in contrast to 0 h (C0 and P0). Genes with log2 Fold Change > 1 or < − 1 and FDR < 0.01 with statistical significance. Heatmap values are rlog-normalized and scale by z-score. Color scale ate top right represent lowest to highest change in expression (blue and red)

The results were also visualized at the level of individual gene expression (Fig. 1). The functional categories of genes up-regulated at 0 h (C0/P0) included genes involved in amino acid metabolism (CTRG_00021, CTRG_00141, CTRG_00079, CTRG_00221, and CTRG_00294), mitochondrial activity (CTRG_00165, CTRG_00269, CTRG_00270, and CTRG_00309), F-actin cytoskeleton (CTRG_00235), sugar transport (CTRG_00112), and enzymes related to the initial fermentation period (CTRG_00301). This suggested that at 0 h, the yeast contaminant expressed more genes related to initial fermentation as ATP formation, energy, cellular cycle, and to start for the expression of genes related to tolerance/resistance to the adverse fermentation condition (Fig. 1a). On the other hand, in the 8 h samples (C8/P8), genes for ammonium metabolism (CTRG_ 00229, CTRG_00119, CTRG_00031, CTRG_00152, CTRG_00040, CTRG_00051, CTRG_00127, CTRG_00013 and CTRG_00254), transcription factors (CTRG_00028, CTRG_00252, CTRG_00025, and CTRG_00253), and stress oxidative (CTRG_ 00011, CTRG_00046, and CTRG_00031) were up-regulated. This may have reflected high metabolic activity of the yeast contaminant, and its adaptation to the fermentation process, coinciding with the peak of bioethanol production. In this step it also highlights adverse effects as high temperature and ethanol concentration (affect the cellular membrane and viability), suppression of nutrients directly linked the viability e metabolic activity the yeast, which are conditions usually observed in 6–8 h of the fermentation process (Fig. 1b).

### GO analysis

**Fig. 2:**
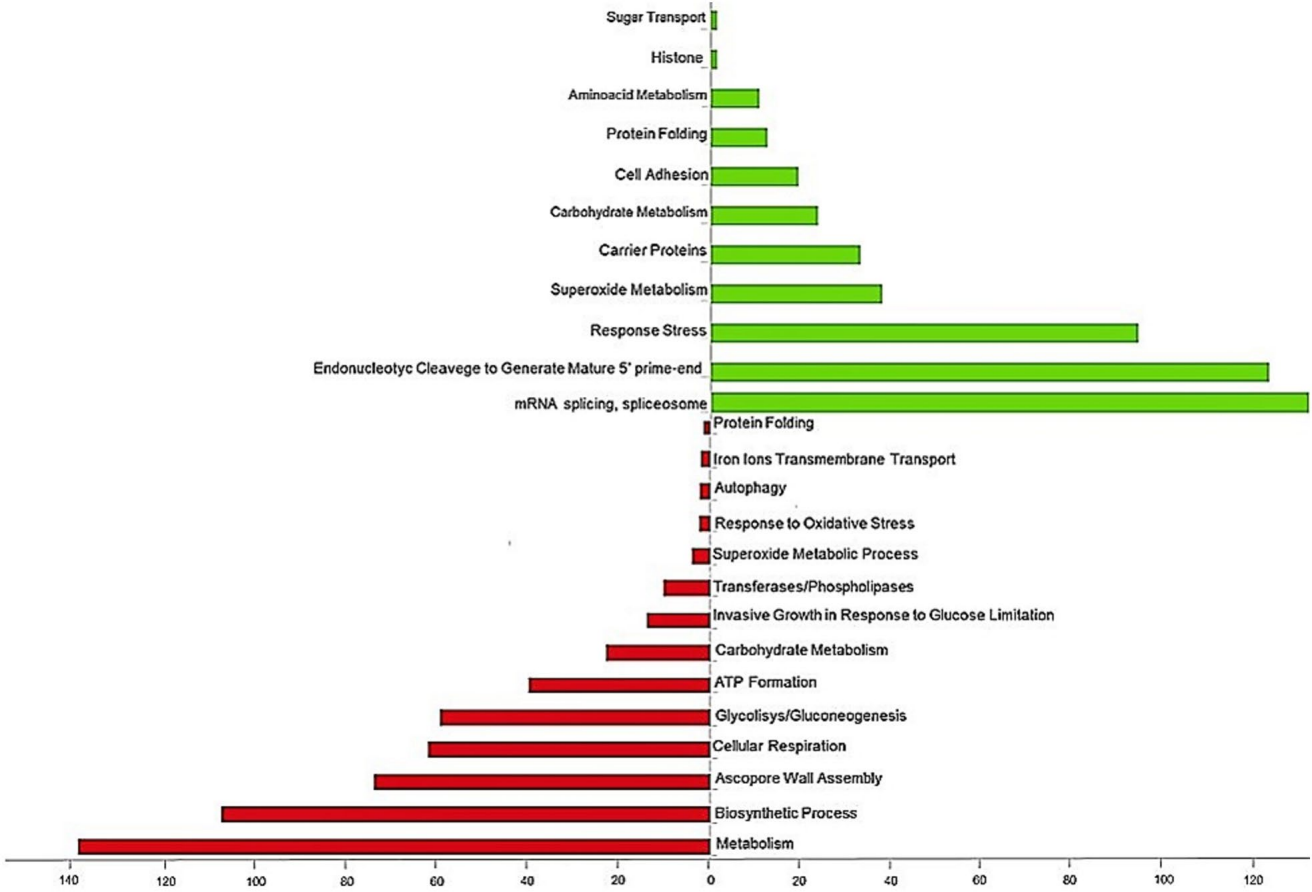
Biological process quantification for up-regulated and down-regulated genes of contaminant yeast, *C. tropicalis* at 0 h and 8 h in the fermentation alcoholic cycle. **a** Biological process represent among down-regulated genes at time 8 h (C8 and P8), represent per the bars in red color in contrast at time 0 h (C0 and P0); **b** biological process represent among up-regulated genes at time 8 h (C8 and P8), represent per the bars in green color in contrast at time 0 h (C0 and P0)

The results of GO analysis corresponded to DEG data, with a high number of down-regulated genes involved in such cellular processes, as enzymatic process of transferases and phospholipases, wall/cell membrane, energy, metabolism, and ATP formation (Fig. 2). Up-regulated genes were enriched in functions related to the spliceosome, ribosomes/transcription, amino acid metabolism, and oxidative stress (Fig. 2).

## Discussion

Bioethanol production proceeds in several stages. The most important factor for the efficiency of bioethanol production is the capacity of a microorganism to consume the entire starter compound to which it is exposed, and to perform the relevant biochemical reactions in a dynamic and coordinated manner. If all the required stages of conversion of sugars, formation of cellular energy and production of bioethanol are complete and synchronized, the production is effective. However, in the Brazilian industry, the presence of contaminants is a problem directly affecting process productivity (Costa et al. 2015). Contaminants found in Brazilian distilleries may be of bacterial or fungal origin, and may reduce bioethanol production by growth inhibition, reduction of nutrient availability, and increasing acidity (Bayrock et al. 2003). The origins of these contaminants include the soil, plant, must, stillage, and yeast cream after centrifugation in batch processes (Cabrini and Gallo 1999; Limtog et al. 2014).

Basílio et al. (2008) pointed out that *C. tropicalis* is one of the most relevant contaminants found in the sugarcane juice from Brazilian distilleries that causes severe contamination and directly impacts production. This was evidenced by the observation that *C. tropicalis* assimilates approximately 63% of sucrose compared to the amount assimilated by the industrial standard yeast strain, *S. cerevisiae* (Basílio et al. 2008). The similarities between these two yeast strains go beyond the phenotypic characteristics and fermentative profiles, and include the transcriptional profile, the analysis of which has revealed correlation between the expressions of many genes in the two species. Genes for the essential nuclear and cytoplasmic functions have been described in *C. tropicalis* (Butler et al. 2009). It is interesting to note that this level of expression and molecular details essential for the production of bioethanol present in *S. cerevisiae* were also observed in the *C. tropicalis* samples analyzed in the current study.

The adaptive response of yeast to fermentation may be associated with the genetic machinery of the cell. Studies identified a locus or a group of genes responsible for adaption to a fermentative environmental would be useful for to verify the level of tolerance to the stress of the yeast during the fermentation process (Stambuck et al. 2009; Zaky et al. 2016). It has been shown that the versatility of yeast, enabling it to adapt and grow under new conditions, is essential for its viability. Similar observations were made for 0 h samples in the current study, confirming that the growth rate increase is associated with cellular homeostasis and cell–cell ratio (Ibañez et al. 2014). Further, the expression of genes from processes important for the initial fermentation stage was higher at 0 h than after 8 h, e.g., amino acid metabolism, assimilation of metals, cellular respiration, and adaptive processes (tolerance).

Amino acid metabolism is essential for the overall metabolism of the eukaryotic cell, since it directly regulates sugar metabolism (Jiranek et al. 1995). Based on the gene expression profiles, assimilation of amino acids was higher at 8 h than at 0 h, suggesting that the yeast contaminant behaved similarly to the standard *S. cerevisiae* strain in terms of fermentation kinetics (Marques et al. 2018). The obtained data corroborated those of Jiranek et al. (1995), and Albergaria and Aneborg (2016), who showed that increasing the number of non-*Saccharomyces* yeasts in the fermentation process increased the capacity to assimilate amino acid sources directly derived from the sugar at the beginning of fermentation, and dependent on innate metabolism and process conditions (Jiranek et al. 1995; Albergaria and Aneborg 2016).

Throughout the fermentation process, a source of nitrogen is required for yeast growth and metabolism (Gobert et al. 2017), and nitrogen deficiency leads to biomass loss and viability loss (Hazelwood et al. 2008). The sources of nitrogen assimilated during fermentation include arginine, valine, asparagine, ammonia, alanine, and glutamine. It has been demonstrated that *S. cerevisiae* prefers ammonia as the nitrogen source (Kensawad et al. 2015). The mechanisms of nitrogen assimilation in *S. cerevisiae* are well known, and revealed by such studies as that of Magasanik and Kaiser (2002). The authors demonstrated the expression of assimilation permeases for preferred nitrogen sources, with the repression and degradation of secondary sources via a control system known as nitrogen catabolite repression (NCR), enhancing fermentation efficiency (Magasanik and Kaiser 2002). However, these mechanisms are not well known in non-*Saccharomyces* species, even though some studies indicate that the mechanism of nitrogen assimilation in a fermentative medium may be similar to that of standard yeasts *S. cerevisiae* (Jolly et al. 2014), as was suggested for *C. tropicalis* in the current study.

We observed the expression of many genes encoding mitochondrial enzymes linked to amino acid biosynthesis, evidencing the potential use of *C. tropicalis* in fermentation. These proteins are located within the mitochondrion, and are involved in the transport of electrons and the formation of ATP via a reduction of NADH and NADPH, an essential step of the fermentation process (Kahar et al. 2017). Of the enzymes important for the biosynthesis of these amino acids, two were highlighted in the current study (delta-1-proline-5-carboxylase reductase and delta-1-proline-5-dehydrogenase), revealing the importance of proline in *C. tropicalis* metabolism (Brandiss and Falvery 1992). Proline, in turn, is a rich source of nitrogen by is converted step of the electron transport chain, as well as other amino acids, resulting in the generation of NADP^+^ (Takagy 2008).

Further, we observed increased expression of genes related to lipoic acid biosynthesis, mainly at 0 h. This suggested that the yeast cell uses all possible pathways for energy formation at that time, to engage in fermentation. Lipoic acid, in addition to neutralizing free radicals, is also associated with energy production (Kursu et al. 2013). In *S. cerevisiae*, lipoic acid has been linked to oxidative decarboxylation reactions via a multi-enzyme complex, which directly affects the activity of the enzymes pyruvate dehydrogenase (PDH) and alpha-ketoglutarate dehydrogenase (KDH), linked to cellular energy generation (Schonauer et al. 2009).

The sequencing data also suggested the activity of the enzyme cytochrome C in *C. tropicalis* at 0 h. This was unsurprising, considering the essential role of this enzyme in ATP formation (Mc Clelland et al. 2014). The enzyme is located in the inner part of on the lipoprotein membrane the mitochondrion, and plays a key role in the respiratory chain, in addition to being an intrinsic activator of the apoptosis (Zhao et al. 2018). In eukaryotes, it is linked to the COX pathway for ATP formation (Garcia-Villegas et al. 2017).

A gene encoding an integral membrane protein belonging to the family of phosphate transducers (PHO89) was also expressed at 0 h, supporting the notion that the yeast was using all viable mechanisms for energy generation at that time. This was consistent with what has been reported for *S. cerevisiae* (Samyn and Persson 2016). This protein exhibits interesting characteristics in eukaryotes: it is induced by calcium levels change (Ca^2+^) in conditions of stress; it is involved in cellular homeostasis; and also acts as a pH sensor of the fermentative medium (Wang et al. 2015). In *S. cerevisiae*, activation of the Ca^2+^ signaling and calcineurin pathways marks an adaptive stress response (Sengottayan et al. 2013). One of the most frequently observed effects of pH change is the alkalization of the growth medium, which negatively impacts the absorption of metals and glucose (Ãrino 2010). In *Candida albicans*, changes in pH also affect yeast morphogenesis and pathogenicity, indicating that the environment is fundamentally important for the cellular wellbeing of eukaryotes (Wang et al. 2011).

The F-actin gene was one of the identified DEGs in the current study, and is directly linked to the fundamental process of endocytosis (Wang and Carlssom 2017). Actin plays a role in cell structure, including cell wall growth, in yeasts as *Saccharomyces* sp. and *Candida* sp. It also plays a role in polarity maintenance and resistance to osmotic forces (Suzuki et al. 1998). In fermenter yeast, actin filaments are present at vacuolar membrane fusions, directed by proteins RhoGTPases (Rho1p and Cdc42p) linked to enhancing cell growth and maintaining cell osmolality during fermentation (Bodman et al. 2015).

Expression of genes acting on the glucose pathway by the activity of permeases was detected at 0 h and 8 h in the current study. This indicates that *C. tropicalis* uses glucose to generate energy throughout the fermentation cycle, keeping other cellular processes active, and thus, competing with standards yeasts *S. cerevisiae* up until end of the fermentation process.

The expression of genes linked to the production of pentose was observed after 8 h. This corroborates the other findings of the current study, because of the link to the structure of nucleic acids, mainly ribose. Indeed, expression of these genes accompanied the increased expression of genes linked to nuclear functions.

Sequencing data for the 0 h sample indicated active functions relating to ATP production and adaptation to environmental stress, and a classical phenotypic of fermenting yeast. By contrast, at 8 h, in addition to the classical functions apparent at 0 h, the expression of nuclear functions linked to histones, spliceosome, splicing, other processes of transcription and cellular division, and the expression of tolerance genes (resistance processes) was enhanced. These observations may be interpreted in the context of viability loss associated with high bioethanol levels at 8 h, since viable cells are in a metabolically active state. The increase in pyrimidine production, transcription, splicing, and spliceosome is important, acting as a rebound effect of the cell loss. Cells produce substantially more proteins (such as phosphatases, redutases, and transferases) to maintain viability and remain metabolically active. Accordingly, Xu et al. (2012) proposed a new strategy of improving the production of pyruvate and reducing the production of metabolic by-products by regulating pyrimidine biosynthesis, as observed in the 8 h sample in the current study.

The above findings are consistent with the observations of Shi (2017) and Scheres and Nagai (2017), who reported that pre-mRNA splicing and the spliceosome act to concatenate junctions, and form mature mRNA molecules for translation. These molecular activities also reflect active fermentation, as the nuclear activities are related to the cell status, once again reflecting the fermentative behavior of the yeast *C. tropicalis* (Zafrir and Tuller 2017).

In light of the presented sequencing data, we conclude that *C. tropicalis* is a potential and relevant contaminant of the alcoholic fermentation process. The data revealed a functional and transcriptional similarity between this contaminant yeast and the standard yeast *S. cerevisiae* used in the industrial fermentation process. The contaminant yeast is versatile, as it expressed genes for adaptation, growth, competitiveness (mainly for nutrients), multiplication, and tolerance of the fermentative process. The data also indicated that in addition to the processes, and activation of the fermentative and metabolic pathways that had been established for *S. cerevisiae* during bioethanol production, *C. tropicalis* adapts to tolerate the adverse conditions of the fermentation cycle (increased temperature, osmotic stress, nutrient level reduction and competition for nutrients, and bioethanol toxicity after 8 h). On transcriptional level, compared to the standard yeast *S. cerevisiae*, some similarities in gene activation were apparent. Interestingly, *C. tropicalis* activated genes specific to bioethanol production and an appreciable number of resistance genes in the first hours of fermentation, with continued expression throughout the entire fermentation cycle. That is different from *S. cerevisiae*, in which genes for bioethanol production are the predominant genes expressed throughout the 8 h fermentation cycle. The presented findings, i.e., the determined behavioral and transcriptional profiles of the analyzed *C. tropicalis* cells during an 8 h fermentation process, can be used to inform future antimicrobial research, to detect and combat *C. tropicalis* contamination during the fermentation process for industrial bioethanol production.
